# Investigating the Unbinding of Muscarinic Antagonists from the Muscarinic 3 Receptor

**DOI:** 10.1021/acs.jctc.3c00023

**Published:** 2023-07-17

**Authors:** Pedro J. Buigues, Sascha Gehrke, Magd Badaoui, Balint Dudas, Gaurav Mandana, Tianyun Qi, Giovanni Bottegoni, Edina Rosta

**Affiliations:** † Department of Physics and Astronomy, University College London, London WC1E 6BT, United Kingdom; ‡ Dipartimento di Scienze Biomolecolari (DISB), University of Urbino, Urbino Piazza Rinascimento, 6, Urbino 61029, Italy; § Institute of Clinical Sciences, University of Birmingham, Edgbaston, B15 2TT Birmingham, United Kingdom

## Abstract

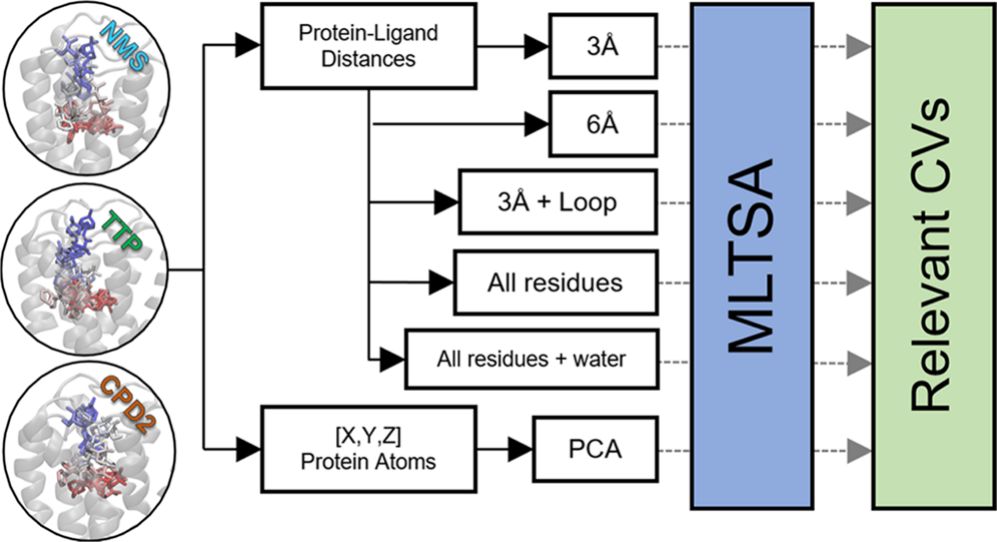

Patient symptom relief is often heavily influenced by the residence time of the inhibitor–target complex. For the human muscarinic receptor 3 (hMR3), tiotropium is a long-acting bronchodilator used in conditions such as asthma or chronic obstructive pulmonary disease (COPD). The mechanistic insights into this inhibitor remain unclear; specifically, the elucidation of the main factors determining the unbinding rates could help develop the next generation of antimuscarinic agents. Using our novel unbinding algorithm, we were able to investigate ligand dissociation from hMR3. The unbinding paths of tiotropium and two of its analogues, *N*-methylscopolamin and homatropine methylbromide, show a consistent qualitative mechanism and allow us to identify the structural bottleneck of the process. Furthermore, our machine learning-based analysis identified key roles of the ECL2/TM5 junction involved in the transition state. Additionally, our results point to relevant changes at the intracellular end of the TM6 helix leading to the ICL3 kinase domain, highlighting the closest residue L482. This residue is located right between two main protein binding sites involved in signal transduction for hMR3′s activation and regulation. We also highlight key pharmacophores of tiotropium that play determining roles in the unbinding kinetics and could aid toward drug design and lead optimization.

## Introduction

Muscarinic receptors (MRs) are a five-membered subtype group of transmembrane receptors, which form an important part of the parasympathetic nervous system. They are activated by neurotransmitters such as acetylcholine and muscarine^1^ and transmit extracellular signals to the cell interior, which makes them attractive drug targets.^2^

The sequence identity between the five MR isoforms is low, except between the transmembrane regions.^3,4^ This region contains seven α helix substructures, which anchor the protein in the outer membrane of the cell.^5^ On the cytoplasmic side, the receptor is bound to a GTP-binding protein, which is responsible for the subsequent signal transduction. Therefore, MRs are part of the G-protein-coupled receptor (GPCR) superfamily.

Downstream signaling can be spontaneously induced when MRs bind to GTP-binding protein, even in the absence of the corresponding agonist.^6^ Activation, as well as the downstream signaling, can be suppressed when suitable antagonists are bound to MRs. This can be exploited pharmacologically,^7^ and several important muscarinic antagonists were developed and used for instance, as bronchodilators in the treatment of asthma or chronic obstructive pulmonary disease (COPD).^8−11^

Human MRs (hMRs) are expressed in a variety of tissues in the human body; therefore, a drug with low selectivity may cause severe complications and side effects.^12^ While the hMR3 isoform—which controls the tension of the smooth muscle tissue in the bronchial tubes—is the actual target of bronchodilators, the off-target binding to the highly homologous transmembrane region of hMR2 is responsible for serious side effects, especially in the cardiovascular system.^12−15^ Due to the high homology between the two isoforms, the binding affinity of most muscarinic antagonists is very similar. For example, the pKi value of the pharmacologically widely used tiotropium for hMR2 is 10.7 and that for hMR3 is 11.0.^12^ Nevertheless, tiotropium shows a high selectivity because the dissociation rate from hMR2 is significantly higher compared to that from hMR3 by about one order of magnitude.^12,16,17^ As a consequence, the residence time of tiotropium in the hMR3 isoform is very long and the binding was considered to be kinetically irreversible.^12,18^

In general, the drug unbinding process is a rare event; it is highly challenging to study it experimentally, and the detailed mechanism is still mostly unknown. However, there are several computational studies available that attempt to approach this problem via molecular dynamics (MD) simulations.^19,20^

Simulations on the β-2 adrenergic receptor using RAMD found two different types of pathways for the unbinding of the β blocker carazolol: one of them along the long axis directly into the extracellular space and one laterally into the membrane.^21^ Recently, it was shown that the path leading directly into the membrane is probably an artifact caused by the force constants of the biasing potentials being too high.^22^ For the same receptor, binding paths for several antagonists and agonists could be identified by conventional MD.^23^ A free-energy profile (FEP) was also presented, which is characterized by two barriers. The first barrier describes the process of docking of the ligand from the solution to the tunnel entrance of the receptor (the extracellular vestibule). The second barrier is on the way of the ligand from the extracellular vestibule to the orthosteric binding site.

Later works using metadynamics and Markov state models (MSMs) found the resting state in the extracellular vestibule to be very shallow and a significant barrier for the desolvation process could not be found.^24^ It is now largely consensus in the available literature that the rate-determining step is indeed on the way from the vestibule to the binding site.^25,26^

Previous studies on the unbinding path of the hMR2 receptor and its agonist iperoxo have also shown that the process encompasses two steps. In these unbinding processes, the rate-limiting step was found to correspond to the ligand exiting from the orthosteric binding site to the extracellular vestibule.^27,28^ However, we note that these assumptions have not been validated yet in simulations on hMR3. Two different exiting pathways are suggested, either with the charged amine moiety of the ligand pointing toward the extracellular space or pointing toward the orthosteric site along the unbinding path. The first one (that we also identified as more favorable in this work) involves the rotation of the ligand and its exit through the extracellular vestibule, while the second one is characterized by the rearrangement of the extracellular loop 2 (ECL2) limiting the ligand from entering the solvated state. Free-energy profiles for the unbinding were estimated using metadynamics; however, calculations of the free-energy barrier or unbinding rates proved to be challenging due to force field inaccuracies.^28^ Given the homology between hMR2 and hMR3, similar limitations are expected to arise, which have been considered for this study.

**Figure 1:**
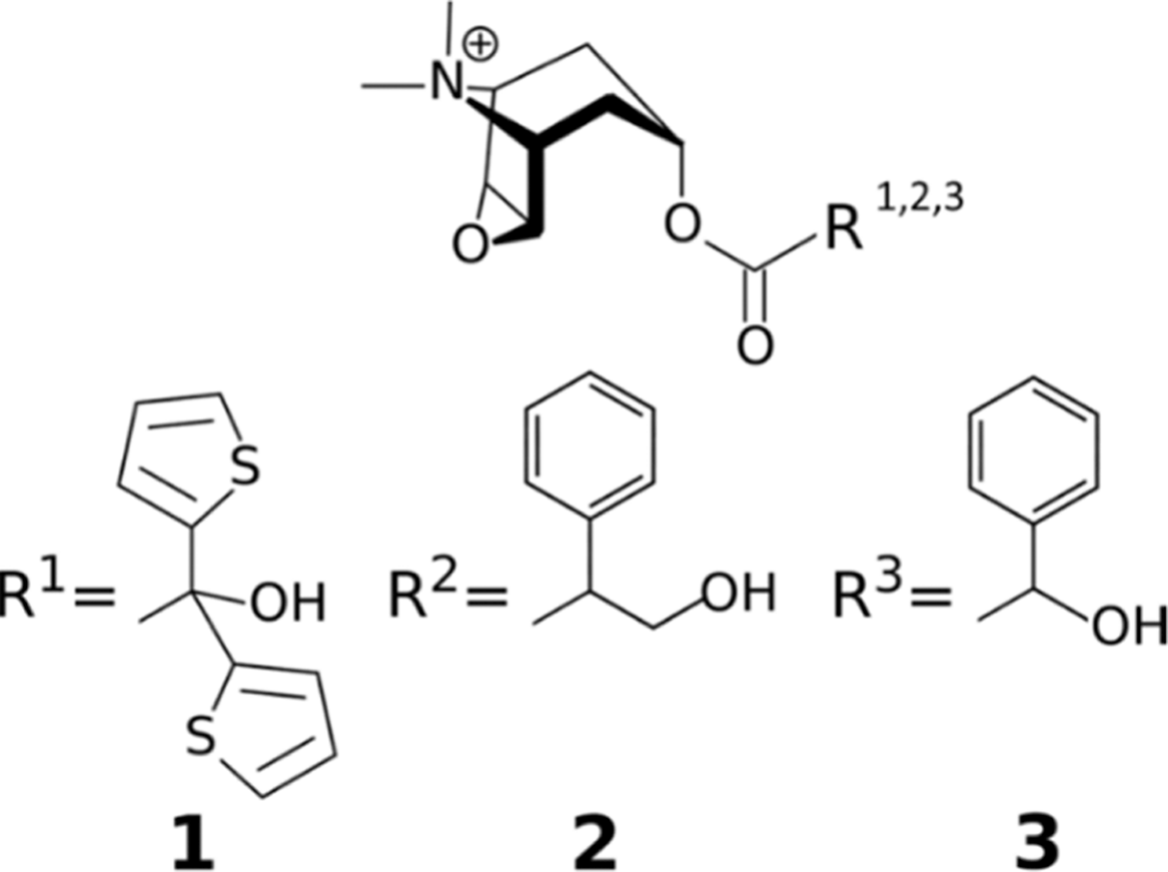
Structures of the ligands investigated in this study: tiotropium (**1**), *N*-methylscopolamin (**2**), and homatropine methylbromide (**3**).

In this work, we applied our recently developed unbinding algorithm^29^ to hMR3 to investigate the dissociation of tiotropium (**1**) and two structurally similar ligands, *N*-methylscopolamin (**2**) and homatropine methylbromide (**3**) (Figure 1). The obtained unbinding pathways were refined using an adaptation of the finite temperature string method.^30^ Finally, the transition state (TS) of the tiotropium unbinding was detailed and analyzed with the aid of machine learning (ML) to identify prominent interaction pairs of the ligand and the receptor at different levels. Additionally, we also revealed key conformational changes of the protein that define the downhill trajectory outcomes.

## Methodology

### Starting Structure

The starting coordinates for hMR3 were obtained using a rat MR3 crystallographic structure, PDB ID 4U14,^31^ with a resolution of 3.57 Å and with tiotropium bound in the orthosteric site. Our structural model was truncated to the transmembrane helices and the extracellular loops, which are highly conserved between human and rat (rat and human sequences share a 91.85% identity for the whole protein and a 97.45% identity for the regions included in our simulations), and contain the necessary and sufficient domains for ligand unbinding.^3^ In our simulations, the T4 lysozyme sequence was omitted.

### Parameterization and System Building

The protein was inserted into a membrane using the membrane builder^32−34^ of the CHARMM-GUI web server^35−37^ and then solvated in water^38^ with 150 mM KCl. The membrane consists of POPC:DMPC:PYPE:DMPE in a ratio of 1:2:3:4, chosen on the basis of earlier studies of hMR3 and on the tracheal membrane tissue.^39^

Initial structures with bound ligands **2** and **3** were generated by manually modifying the R group of tiotropium in the original structure (Figure 1). Both *N*-methylscopolamin (**2**) and homatropine methylbromide (**3**) are chiral compounds; the S-enantiomer for **2** and the R-enantiomer for **3** was used considering their clinical relevance [DrugBank DB00462 and DB00725]. The ligands were geometry-optimized at the B3LYP/6-31G** level of theory,^40^ applying the ORCA 4.1 software suite.^41−43^ With the optimized structures, force field parameters for the ligand were defined using the CHARMM-GUI ligand reader.^44^

The all-atom CHARMM36m force field was used for the proteins,^45−48^ lipids,^49,50^ and the TIP3P model^38^ for the water. Simulations were carried out with the NAMD software package^51^ using input generated by the CHARMM-Input generator.^52^ The cutoff for nonbonded interaction was kept at 12 Å, and the switch distance was at 10 Å. Electrostatic interactions were handled by a particle-mesh Ewald solver with a grid spacing of 1 Å. The temperature was kept at 310.15 K using Langevin dynamics. The pressure was kept at 1.013 bar by the Nosé–Hoover Langevin piston pressure control.^53,54^

The structures were first energy-minimized according to the CHARMM-GUI scheme and subsequently equilibrated for 50 ns.

Classical MD simulations on the apo system without any bound ligands were also performed for comparison. The same protocol was applied as detailed above for the construction of the apo system. Three 10 ns long production runs were then performed. The overall root-mean-square deviation (RMSD) with respect to the initial crystal structure demonstrated a well-equilibrated system in both the apo and the ligand-bound states during the 10 ns long production runs (Figure S1).

### Unbinding Simulations

The unbinding procedure was followed as described in our previously published^29^ protocol. After the equilibration, a 20 ns production run without any restraints was performed. During this production run, all interacting pairs of heavy atoms—one in the ligand and one in the protein—were identified. Thereby, a pair is defined as “interacting” if the distance between the atoms is below 3.5 Å for more than 50% of the simulation time. Based on the sum of these interacting distances, a collective variable (CV) is defined and restrained harmonically.^29^ During an iterative process, subsequent simulations of 10 ns use this biasing CV with a force constant of 10 kcal mol^–1^Å^–2^. The constraint position (i.e., the length) of the CV is monotonically increased. In the next iteration, new interaction sites are identified in the same way as before and these are added to the CV. Interactions are discarded and removed from the CV if the distance between the atoms is larger than 11 Å. A shorter cutoff distance results in the ligand falling back into the original binding position after a few iterations. This procedure is repeated until the ligand is displaced out of the receptor.

The unbinding simulations were run for 25 iterations, adding up to a total of 240 ns simulation length. Thereby, a total of 52, 50, and 44 interacting protein–ligand distances were identified by our unbinding method along the paths for ligands **1**–**3**, respectively.

### Refinement of the Path Using the String Method

The unbinding path was used as a starting point for the following refinement using the finite temperature string method.^55^ Since the string iterations are computationally very expensive and at the same time converge rather slowly due to the many dimensions, only 20 iterations were calculated.

### Approximation of the TS Region

**Figure 2:**
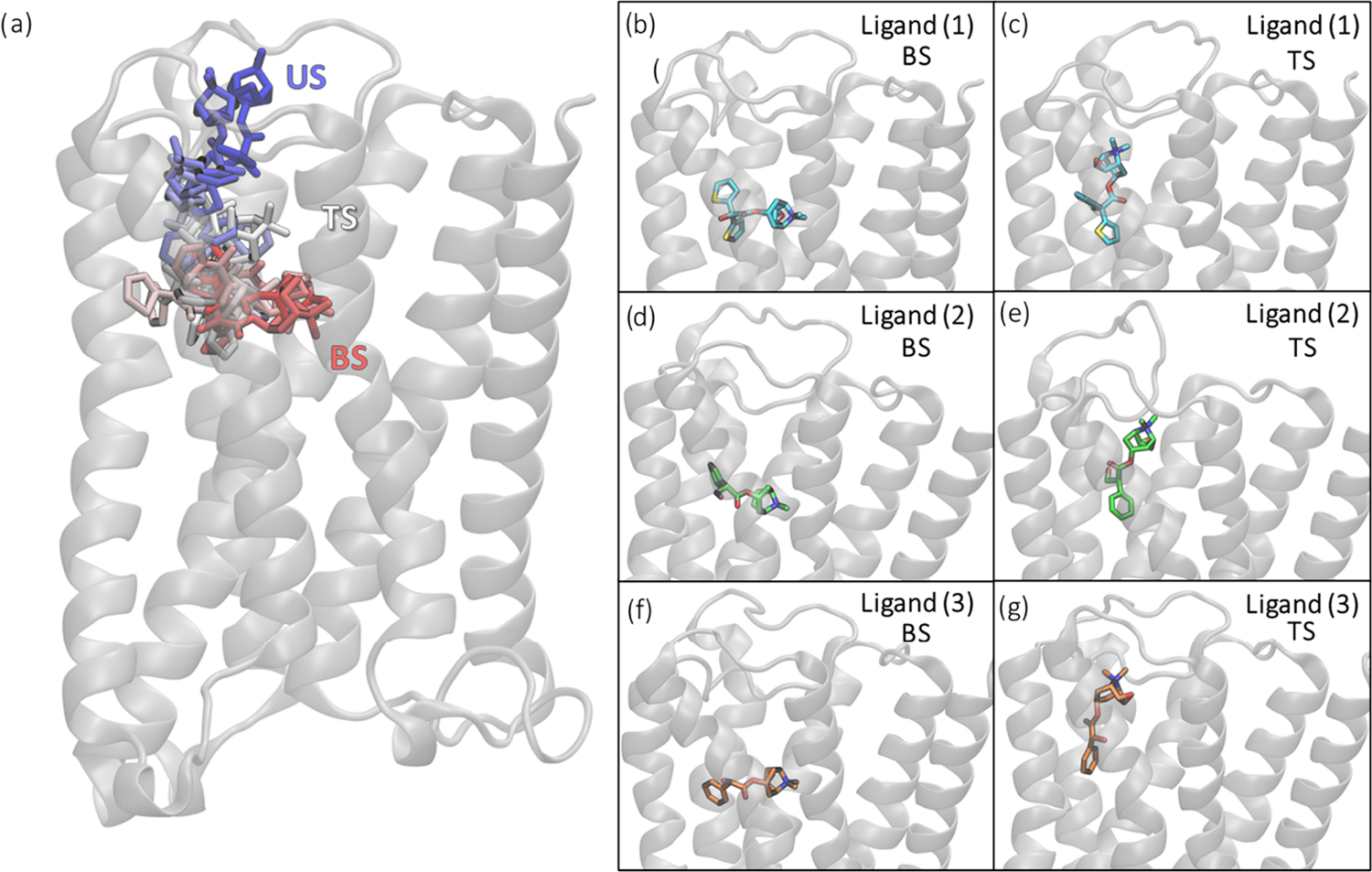
Left: (a) overlay of the structures from the unbinding path of ligand **1** through time starting from the bound state (BS, in red) toward the unbound state (US, in blue) passing through an approximated transition state (TS, in white). Right: stick representations of the three unbound ligands on their original BS (b, d, and f for ligands **1**–**3**, respectively) and their TS (c, e, and g for ligands **1**–**3**).

To approximate a TS structure from the string windows, we identified a set of structures from the string windows, which are very similar in the unbinding paths of all three investigated ligands (Figure 2). We selected five windows as starting points around the window with these distinct structures for ligand **1** and performed 50 independent unbiased (downhill) MD simulations with a 5 ns length each. Thereby, we were able to identify the structure that provided the closest 1:1 ratio of a binding (IN) or unbinding (OUT) event, which we considered to be the TS of the unbinding process. Our TS is reached after the previously existing H-bond is finally broken between the ligand and Asn507.

### Machine Learning Transition-State Analysis (MLTSA)

To aid the identification of the main CVs driving the system across the TS and to pinpoint novel descriptors that determine the fate of a binding/unbinding event, we used our MLTSA.^29^ In this approach, we train an ML model to predict the outcome of downhill simulations with data close to the TS. Subsequently, we make use of the trained models to discover the key TS-defining features of the system.

### Creation of the Data Sets

Using ligand **1**′s identified TS structure as the starting point, we ran multiple 5 ns long unbiased simulations. We classified and labeled 149 downhill trajectories by considering a linear combination of 52 distances to identify which simulations arrive at an IN or an OUT state. A minority of additional trajectories not reaching clearly either the IN or the OUT states after 5 ns were discarded. To train ML models, we created several sets of features containing different distances (CVs) along the simulation frames. To assess intraprotein interactions, a first data set (XYZ-PCA set) included the Cartesian coordinates of all protein atoms (∼6k, not including hydrogens). To reduce the dimensionality, we applied principal component analysis (PCA) and used only the top 100 components as features. To enable more interpretable localized features, we created further data sets containing ligand–protein distances. The first such set (3 Å set) contained all interatomic distances between the ligand and the protein within 3 Å of the ligand at the starting TS position, excluding hydrogens. The second data set of this type (6 Å set) was created in a similar fashion to the previous one but with a cutoff of 6 Å instead. For the third data set (3 Å + ECL2/TM5 set), the same data was used within 3 Å of the ligand, with the addition of the interatomic ligand–protein distances of the extracellular loop 2 (ECL2) and the transmembrane region 5 (TM5), including residues from I222 to T231. An additional data set, to assess overall ligand–protein contributions, was also created (allres set), which considers all residues and includes the closest distance between the residue and the ligand at each simulation frame. This data set was also amended with the closest 8 water molecules; their distances to the ligand (allres + wat set) were included to enable the assessment of the role of water molecules.

### Machine Learning Models and Training

We used two different ML models: a multilayer perceptron (MLP) neural network classifier^56^ and a gradient boosting decision tree (GBDT) classifier.^57^ Both models were trained to predict the outcome (IN/OUT) of the simulations from early on data at the time range from 0.05 to 0.1 ns, totaling 2500 frames per simulation. We trained 100 independent MLP and GBDT models randomly assigning the 149 simulations into training data (70%) and validation data (30%). Details on the trainings and hyperparameters can be found in the Supporting Information (SI) section ML Models.

### Feature Analysis

**Figure 3:**
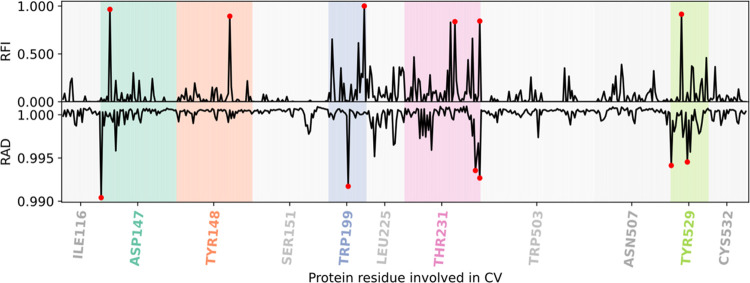
Relative feature importance (RFI, top) and relative accuracy drop (RAD, bottom) are shown for every interatomic distance between ligand **1** and hMR3 in the 3 Å data set. Distances are ordered and clustered by residue number. Residues with the top six distances (red symbols) are highlighted.

**Figure 4:**
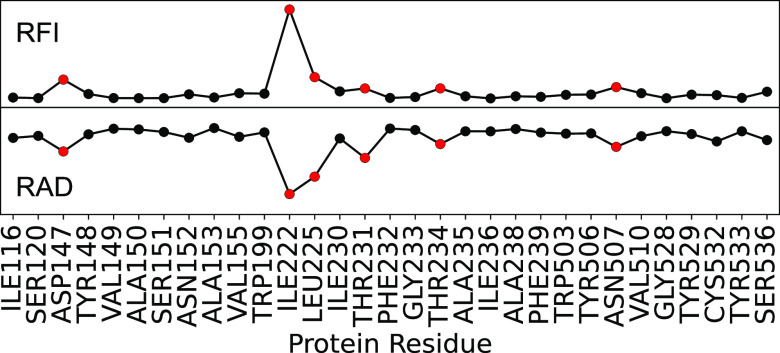
Average RAD (from MLP) and RFI (from GBDT) of the interatomic distances of the ligand **1** per protein residue for the 6 Å data set. In red, the top 6 residues detected by both approaches.

**Figure 5:**
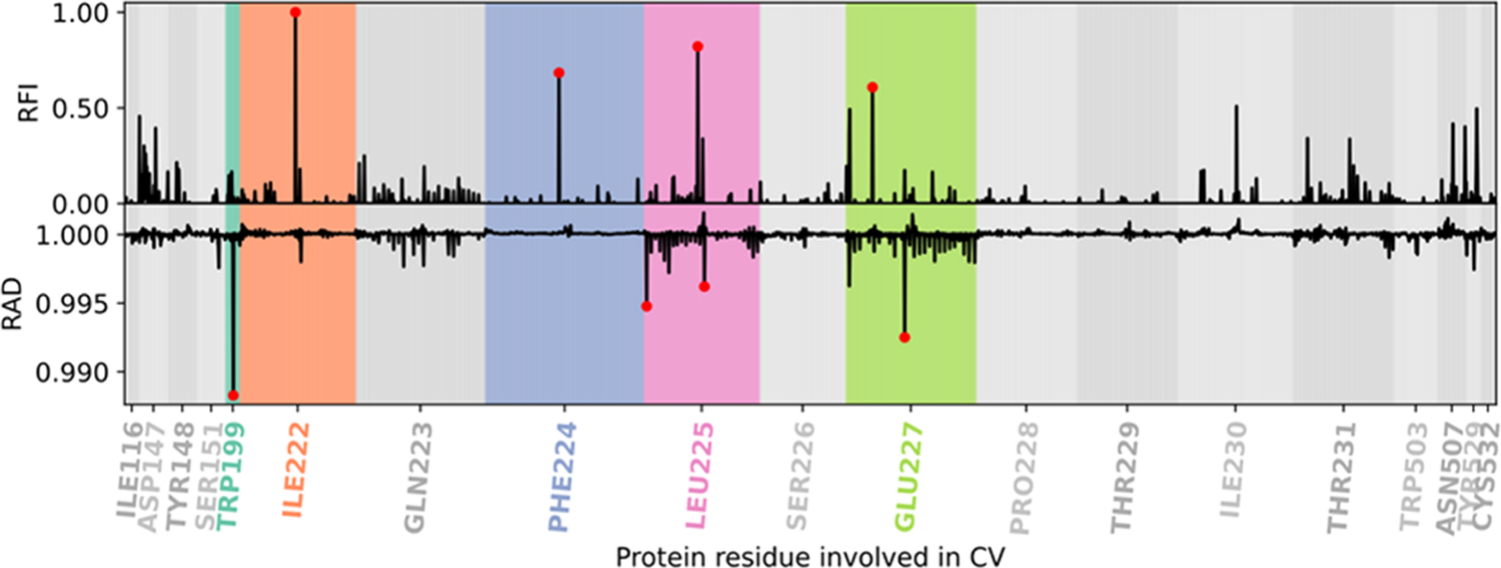
Relative feature importance (RFI) (from the GBDT model) and relative accuracy drop (RAD) (from the MLP model) values for each interatomic ligand–protein distance per residue in the ligand **1**′s 3 Å + ECL2/TM5 data set. Marked in red are the top distances for each model. The most important residues for the ML models are highlighted.

We used the Gini feature importance^58^ to evaluate the relevance of the features from the GBDT models, averaged across the 100 trainings to calculate their relative feature importance (RFI). To identify key features in MLP models, we removed the variance from each feature one by one^29^ and assessed the accuracy drop they encounter when predicting outcomes with the trained models. If the accuracy of the prediction is greatly reduced when a feature is altered, the feature was considered important for the description of the TS. We identified the overall top features averaging the relative accuracy drop (RAD) from all 100 trainings on all data sets used (Figures 3–5).

## Results and Discussion

### Bound State in the Orthosteric Site

In all three ligands, the initial ligand positions in the unbinding simulations are close to the starting bound pose: the charged end of the molecule is nestled in an aromatic cavity, which is formed by the residues W503, Y148, Y506, and Y529. The tyrosines form a cap around the ligand. Simultaneously, the S151 residue coordinates the epoxide group via a hydrogen bond and the negatively charged residue D147 neutralizes the positive charge of the ligand. At the opposite end of the molecule, the N507 residue stabilizes the molecule by a hydrogen bond with the OH group. The same binding mode was also described in recent works.^18,25,31,59^

### Departing from the Binding Site

As illustrated in Figures 2 and S6, the first movement from the binding state (Figure S6A) is a rotation of the charged end of the molecule. Thereby, the hydrogen bond of the epoxide group with S151 is broken and the ligand slightly gains flexibility. Apart from that, the ligand’s position in the binding site remains nearly unchanged (Figure S6B). This first movement is most pronounced for ligand **1**, which follows a helical motion along its longitudinal axis and thus it detaches itself from the aromatic cavity.

This shift is present but less pronounced for ligand **2** (Figure S7a). Subsequently, ligand **2** breaks through the tyrosine-formed ceiling via a path associated with significantly more dislocation of the residues Y148, Y506, and Y529.

In the path of ligand **3**, the entire molecule does not shift; instead mainly the end of the ligand with the thiophene ring moves (Figure S7b). This allows the charged end to slip outwards the aromatic cage in a rolling motion.

### Through the Bottleneck

The new position after the shift allows the molecules to rotate their charged end by 90° toward the direction of the receptor tunnel’s exit (counterclockwise), without exerting a lot of tension on the tyrosine residues forming the aromatic cap. During this rotation, all three paths pass through a state (Figure S6D), which is highly similar in all unbinding trajectories. Interestingly, this rotation was observed to proceed clockwise for the iperoxo ligand unbinding path in hMR2.^28^ This movement positioned the charged end of the molecule pointing toward the membrane in these previous simulations and not to the extracellular vestibule observed by us. We subsequently found by unbiased simulations starting from this structure that either the ligand is led back into the binding site or it moves along the exit tunnel toward the extracellular vestibule (Figure S6E). Therefore, this position can be identified as a TS of the unbinding from the orthosteric site. The pathway is also similar to the previously reported forced dissociation with acetylcholine as well as tiotropium (**1**) on hMR3 (Figure S8) and with a slightly tilted orientation on hMR2.^25^ In addition, the studies of Galvani et al.^60^ and Capelli et al.^27^ suggest the existence of an alternative orientation of tiotropium (**1**) on its path with its charged amine moiety pointing backward to the orthosteric site. However, we have not observed this in our simulations.

### To the Extracellular Vestibule

After 25 iterations of unbinding simulations, we assessed the final ligand positions and found that it fully unbound from the orthosteric site. The RMSDs of the ligand heavy atoms between the initial and final conformations are 10.1, 11.9, and 10.8 Å for 1, 2, and 3, respectively. Nevertheless, the simulations were not sufficiently long to observe the complete unbinding of the ligand and fully break all contacts with the protein (there are still existing interactions with the ECL2 region and other protein residues). However, as the last step to the full unbinding is thought to be facile and not rate-limiting, our downhill trajectory outcomes are already assessed by our analysis to identify IN and OUT trajectories, and none of the analyses is affected by these remaining interactions. In line with the consensus literature, it is estimated that the final unbinding step from the extracellular vestibule has a significantly lower barrier; therefore, it does not likely contribute to the off rate.^24,25^

### Downhill Trajectories from TS Structures

We evaluated starting structures from 5 string windows near the bottleneck conformations. The structure closest to the TS position led to 85 and 64 downhill trajectories of 5 ns reaching the IN and OUT states, respectively (Figure S9). To explore the time range where the TS is probed, we performed initial ML trainings to identify the region where the ML method can accurately, but not with full confidence, predict the final outcomes from as early timeframes as possible. We found that this was already possible from 0.05 to 0.1 ns timeframes. Trainings at different times can be found in Figure S11, and final accuracies for all data sets are listed in Table S1.

### Assessing Contributions from Protein Conformational Changes

To consider changes in the protein structure affecting the unbinding, we analyzed the protein Cartesian coordinates via their top 100 PCA components (Figure S10). We were able to predict the outcome very accurately, obtaining average test accuracies of 100% (MLP) and 93% (GBDT). Out of the 100 components, the first two PCA components were important for both RFI and RAD. Additionally, PCA23 and PCA59 were important for RFI (see SI Section S2). The main PCA component represents large-scale movements from the TM2, TM3, and TM6 to TM7 helices, including some ECL1 residues (Figures S12 and S13). The residues that contributed the most are from the middle of TM6, close to the ligand. The second main PCA component (top RAD feature) represents motions from the rest of the protein, mostly from TM4 to TM5, with the ECL2 loop being especially relevant. The largest contributions come from residues (W206, Q207, I222, and Q223) that belong to the ECL2/TM5 junction, some from TM4 that are close to the ligand (I194 and V193). However, due to the broad distribution present in the PCA components, their interpretability is limited. Therefore, we next focused on feature sets that are precisely localized and able to assess specific ligand–protein atomic distances instead.

### Key Feature Identification from the 3Å Data Set

We created a high-resolution data set, which contained atomic distances between the ligand and protein residues within 3 Å of the TS structure of the ligand. Using our 3 Å data set, we achieved a prediction accuracy of ∼78% with MLP and ∼77% with GBDT and obtained consistently similar key features by RAD and RFI (Figure 3). Both models (MLP and GBDT) agreed on the importance of four out of six top residues: D147, W199, T231, and Y529.

Three of these key residues were previously known to play important roles in the unbinding process. D147, as mentioned earlier, interacts with the charged amine moiety in the bound form. Similarly, Y529 is part of the aromatic cage around the ligand. Additionally, the aromatic substructures of the ligand are known to interact with a hydrophobic region close to W199. Mutational studies show an accelerated dissociation for Y529A and reduced half-life for both W199A and D147A, further suggesting their involvement.^18^

Interestingly, T231 was not previously reported and validated as relevant for ligand interactions in the bound state.^18^ Even though there are no experimental studies, it was previously identified computationally to form relevant contacts during the forced dissociation of tiotropium.^25^

### Contribution of the Extracellular Vestibule within 6 Å

To assess the contributions from more distant atoms beyond 3 Å, we also analyzed results from a data set that includes ∼5000 interatomic distances within a range of 6 Å from the ligand at the TS. In this data set, we analyzed both individual feature importances (Figure S14) and average importance values for each residue (Figure 4). Accordingly, D147 and T231 are again part of the top 6 key residues both measured by RAD or RFI. Newly identified key distances include I222 and T234, which were not part of the previous data set, as well as additional heavy atom distances from L225 and N507. L225 was previously reported as relevant for the binding/unbinding kinetics in hMR2/hMR3 experimental studies but insufficient alone to explain the difference between both receptors. N507 is a previously validated relevant interaction that accelerated the dissociation of tiotropium when mutated to Ala (N507A).^18,25^

**Figure 6:**
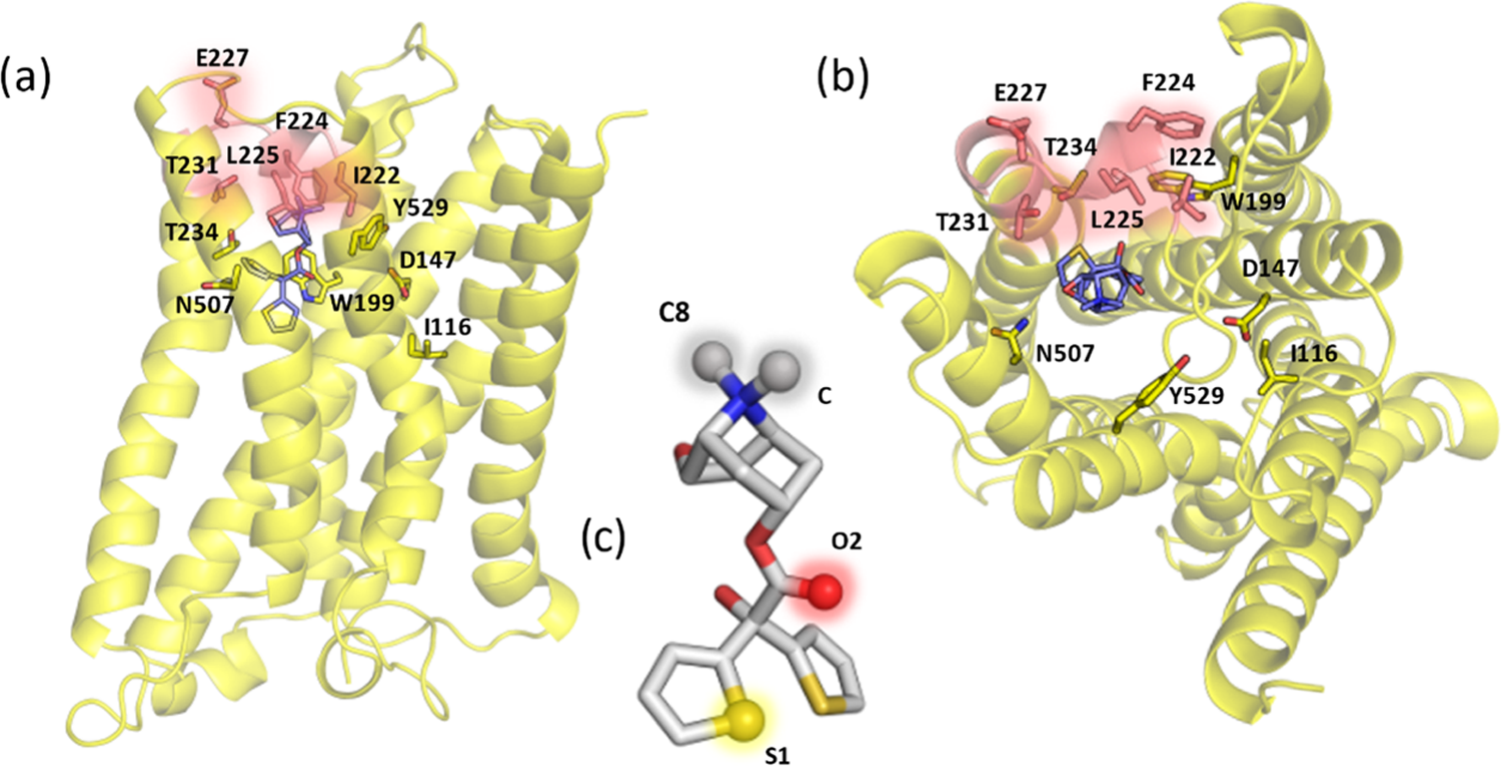
Top: front (a) and top (b) views of the M3 receptor at the TS; the most relevant residues for the unbinding process found by the ML models are shown in sticks. The residues belonging to the ECL2 loop are shown in salmon, which is found to be the most relevant region. (c) Ligand **1**′s structural representation with the most relevant atoms found by the MLTSA, highlighted, and annotated.

**Figure 7:**
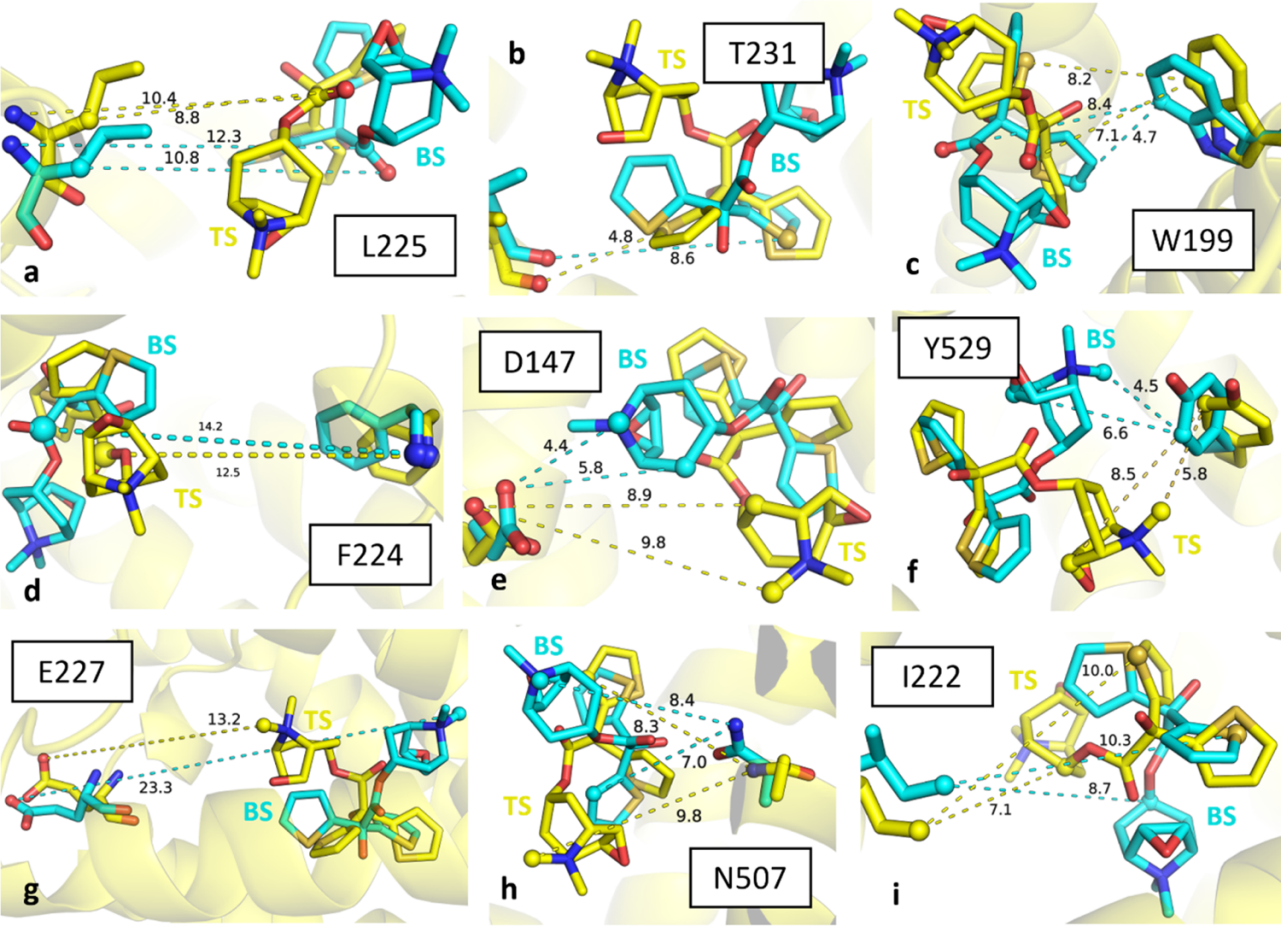
Panels (a–i) are the top nine residues represented as sticks with their protein–ligand (hMR3-ligand **1**) distances consistently found to be the most important throughout the MLTSA analysis across all data sets. The ligand–protein complex at the TS and their distances are shown in yellow; the ones corresponding to the complex at the BS are shown in cyan. The atoms that the interatomic distances represented correspond to are represented as spheres.

**Figure 8:**
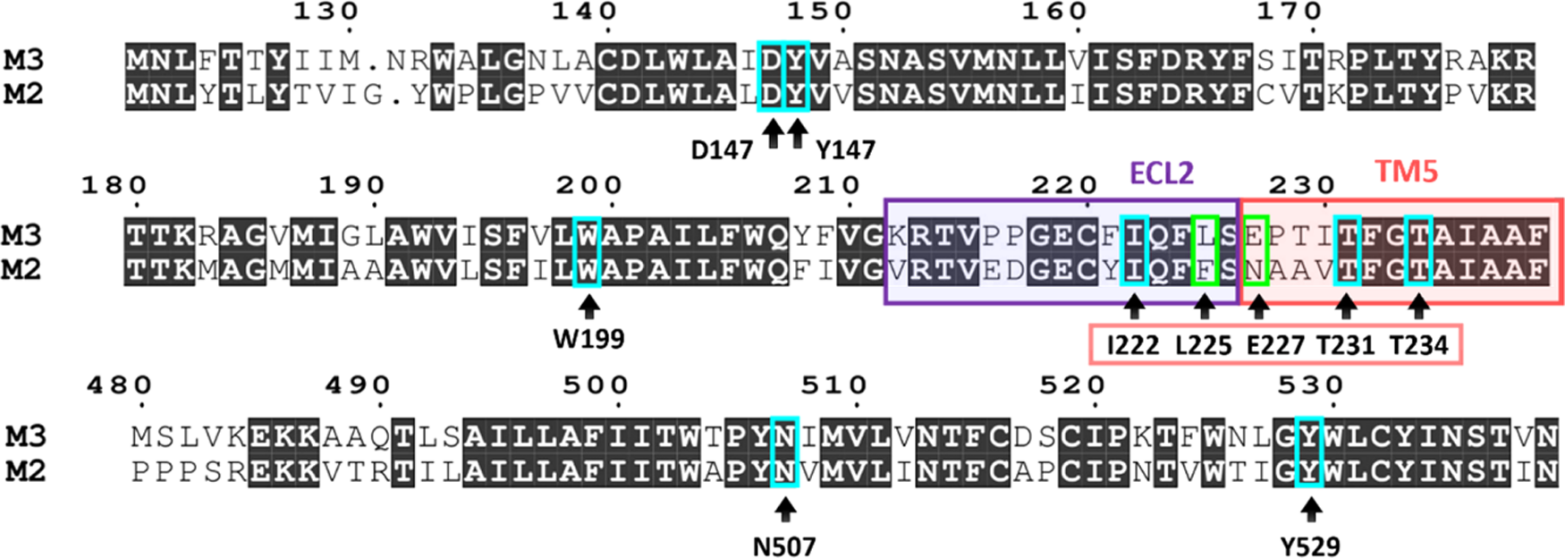
Protein sequence alignment of hMR2 and hMR3 for selected regions involved in the unbinding process. Key residues identified by MLTSA are distinguished as conserved (cyan) or nonconserved (green) between the two receptors. The ECL2/TM5 region is also highlighted (purple and salmon).

Interestingly, the residue with the most relevant interactions is I222 and it was not described previously. Together with L225 and T231, I222 forms a hydrophobic cluster on the extracellular vestibule (Figures 6 and 7a,b,i). The fact that the most relevant residues (I222, L225, T231, T234) are close together in an extracellular loop (ECL2) may be indicative of the importance of this loop for the unbinding. When aligning hMR2 and hMR3 protein sequences, most of the sequence is identical, but the region prior to T231 (ECL2/TM5) has a high genetic variability (Figure 8). Interestingly, preceding I222, there is another variation in the sequence for ECL2: F221 in hMR3 is substituted with Y177 in hMR2. Moreover, this residue is a potential phosphorylation/modulation site for hMR2^61,62^ and thus thought to be not only an important region for allosteric regulation but it could alter the observed unbinding kinetics depending on the phosphorylation state of hMR2. This suggests that the residues between I222 and T231 may be relevant to the significantly different behavior observed between hMR2 and hMR3 in terms of residence times.^12^ Hence, a third data set (3Å+ECL2/TM5 Loop) was created containing all of the residues prior to T231, which range from I222 to T231.

### Exploring the Role of the ECL2/TM5 Junction

In the presence of distances from this region (Figure 5), the top features belong mostly to the ECL2/TM5 junction, except for W199 when using RAD. In hMR2, L225 corresponds to a Phe residue (Figure 8), which is bulkier. Interestingly, this change was previously reported to remove a pocket in hMR2, which is present in hMR3.^25^ The negatively charged E227 is replaced by a neutral Asn in hMR2. Remarkably, both ML models found E227 important, despite its longer distance (Figure 7g). This residue has been mutated to Ala (E227A) previously, resulting in a slight decrease in the half-life of tiotropium, **1**, from 24.5 to 20.1 h. The RFI, however, found an additional key distance involving F224 as one of the most relevant distances. When mutated to F224A, the half-life of **1** is reduced by ∼50% to 13.8 h.^18^

Additional tests with distances from an alternative loop, ECL3, were also added to the 3 Å data set and analyzed (Figure S15) for comparison. These demonstrate no significant contributions from this region, thus validating the unique role of the ECL2/TM5 junction.

### Structural Spotlights of Tiotropium Involved in Unbinding

Our results point to key atomic contributions from only a few selected atoms of tiotropium (Figure 6c). The most prominent moiety corresponds to the methyl groups (C and C8 atoms) that are bonded to the charged amine. Of key relevance is also the S1 sulfur atom from only one of the two thiophene rings, showing key interactions with W199, I222, and T231 (Figure 7, panels c, b, and i, respectively). Finally, the O2 atom from the carbonyl oxygen of the ester group is also important, as identified in interactions with W199, L225, and Y529 (Figure 7, panels c, a, and f, respectively). In agreement with our results, previous studies have shown that the tiotropium analogues with the closest *K_i_* values have a pattern containing all three groups: an amine cap, the carbonyl group in between, and two aromatic rings (thiophene or not) at the end.^18^

### Overall Residue–Ligand Contributions

**Figure 9:**
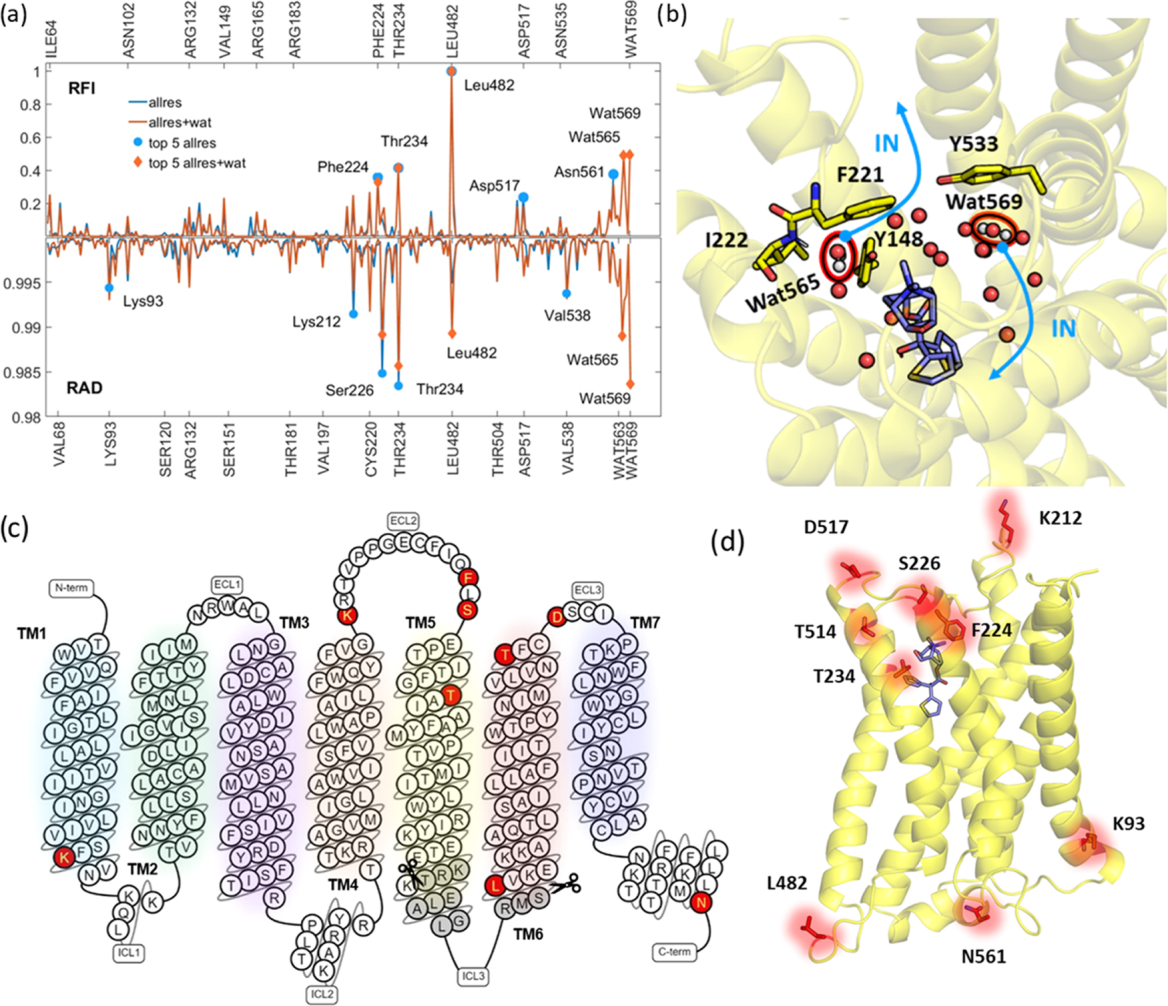
(a) RFI and RAD for the allres (blue) and allres + wat (orange) data sets; highlighted are the top 5 residues for each approach (blue circle and orange diamond, respectively). (b) TS snapshot showing the top two water molecules as well as nearby residues as sticks in the allres + wat data set. The blue arrows highlight the displacement of the water molecules upon re-entering of ligand **1** in the binding site. (c) Diagram representation of the sequence of hMR3 portraying the different secondary structure motifs. In red, the top residues found decisive for the outcome by our MLTSA. In gray, the residues (kinase domain) not included in our simulation system. (d) Top important residues from MLTSA highlighted in the three-dimensional (3D) representation of hMR3, mostly corresponding to the ECL2/TM5 junction and the different ends of the α helices throughout the receptor.

To assess all of the residues in the protein, we decreased the resolution of the feature space and evaluated only features defined via the closest distances between each residue and the ligand (allres data set). This allows us to evaluate all residues, including the ones far from the ligand, which can nevertheless have a key impact on the simulation outcome. The resulting training from this data set yielded ∼79% for GBDT and ∼77% for MLP on their test set. T234, highlighted in our previous results as a key residue in the 6 Å set as well, is the most important feature for RAD and the second most important for RFI, validating its key role (Figure 9a).

A more distant residue that shows key importance is L482, ranked 1^st^ for RFI and 5^th^ for RAD. This distant residue is at the N-terminal end of the TM6, located very near the kinase domain of ICL3, at the interface of the membrane and the intracellular matrix (Figure 9c,d). This could signal changes in the ligand-bound state to the ICL3, which is not modeled in our simulation system. Accordingly, this region is located between two main binding regions of hMR3 for activation and regulation.^63,64^ Pyrophosphatase-2 (PPase 2A), a transmembrane enzyme, which targets the C-terminal region of the ICL3, the “**KRKR**” motif in (“IT**KRKR**MSLIKEKKAAQ”), is thought to be involved in hMR3 dephosphorylation.^65^ Additionally, the muscarinic receptor signaling regulator, SET, a PPase 2A inhibitor, also binds to the same motif.^66^ Furthermore, it was also suggested that protein kinase G II (PKG-II) activates hMR3 via a cGMP-dependent phosphorylation at S481 (“MSLIKEKK” motif).^63,67^ Therefore, this region is thought to be a putative phosphorylation site just preceding L482.^63^ Interestingly, ligand-dependent phosphorylation of S481 was also connected to enhanced dimerization and/or oligomerization.^68^ This has been suggested previously in conjunction with homologous GPCRs,^69−71^ pointing to a general signaling mechanism in this family of proteins.^72,73^ Homo or heterodimerization of kinase domains is often an observed functional requirement along with phosphorylation when activating signaling pathways in general.^74,75^ L482, however, is the first residue in our simulation model after the missing kinase domain; hence, the precise role of signal transduction from the orthosteric site to the ICL3 kinase domain remains to be explored in more detail.

Other key residues also include distant locations that are near the ends of helical domains, similarly to L482: K93, K212, T514, D517, and N561. Some of these residues were identified as important by mutational studies, such as K212V and D517A, that decrease the tiotropium residence time in hMR3. Near T514 and D517, C519 was also previously identified as a key residue for RFI in PCA components 23 and 59.

The ECL2 loop remains key in this data set as well; besides T234, F224, and S226 are also highlighted (Figure 9a). This is validated by the F224A construct, as mentioned earlier, where the half-life of **1** is halved.^18^ In summary, RAD and RFI show a consistent picture, pointing to the key relevance of the ECL2/TM5 junction, in agreement with our previous results.

### Water Plays a Role in the Unbinding Path

Solvent molecules are known to play a crucial role in ligand unbinding kinetics.^76−80^ By both enabling the favorable electrostatic environment and orchestrating movements via hydrogen bonding, water molecules play a role that is often difficult to elucidate. To explore the role of water during the unbinding process, we included the 8 closest water–ligand distances together with the allres data set as additional features. We found a modest increase in both MLP and GBDT prediction accuracies (∼81 and ∼79%, respectively). With these additional sets of features, both RAD and RFI ranked the same two water molecules (Figure 9a,b, labeled 565 and 569) within the top 5 features. L482 remains ranked 1st for RFI and it is 5th for RAD. Both approaches consistently find L482, T234, F224, and S226 as the most relevant together with water molecules (Figure 9c,d). This finding suggests that movements of water molecules in the pocket are also decisive to ligand unbinding in addition to the residues highlighted previously.

Water 565 is located near the ECL2 residues F221 and I222 and forms H-bonds with Y148 and the backbone of I222, part of the ECL2/TM5 junction we highlighted throughout this work. Upon analyzing the most likely distances for IN and OUT trajectories, we observed that this water gets displaced in most of the trajectories as the ligand enters the orthosteric site. On the other hand, water 569 is on the other side of the ligand, closer to Tyr533, as well as Tyr529, which also forms the tyrosine cage. It only partially forms H-bonds with other water molecules, and it is located near a hydrophobic region of the pocket. While for OUT trajectories, this position is not likely to change significantly, for IN trajectories the water moves deeper into the binding pocket as the ligand moves down into the orthosteric site.

### Apo Simulations

To compare the solvent behavior in the active site, we performed additional MD simulations of the apo system and compared it to the ligand-bound state. Interestingly, we found that in the apo system, a potassium ion descended deep into the distal (intracellular side) of the orthosteric pocket. A monovalent cation is present at this site in many structures of class A GPCRs (e.g., in PDB structures 7UL2, 6ZDV, 6WQA, 6TQ4, or 6PS7); a sodium ion at this site is suggested to have a role as a cofactor or as a negative allosteric modulator in signaling.^81,82^

### Water Residence Times

To further compare the mobility of the above-discussed water molecules (Figure 9b) and other water molecules in the binding site, we analyzed the water occupancies. There are considerably more water molecules in the orthosteric site in the apo-form compared to that in the ligand-bound forms, as expected due to the space the ligand occupies. Yet, we found that the extracellular and intracellular bulk water reservoirs do not connect through the receptor in the apo-form (Figure S2A), similarly to the crystal waters observed in experimental structures (e.g., in PDB structures 8CU7, 7PX4, and 6ZG4). However, this bottleneck region (shaded area in Figure S2A) is occupied with water in the ligand-bound state, and the intra- and extracellular water molecules are only separated by the ligand (Figure S2B).

We identified several water molecules in close proximity to the bound ligands. The positions of two such water molecules were located close to the ones that are highly correlated to ligand movements around the TS as discussed above (Figure 9a,b). Accordingly, we analyzed the water occupancy of the H-bonding sites at Y148 (at the hydrogen of its hydroxyl group), C532 (at its backbone oxygen), and S536 (at the oxygen of its hydroxyl group) (Figures S3–S5, respectively). Y148 is located above the bound ligands (closer to the extracellular space) if the ligand is at the orthosteric binding site, whereas C532 and S536 are buried deeper under the bound ligand. Each of these sites is occupied for over 93% of the duration of the ligand-bound and apo production runs and forms H-bonds with different water molecules. Due to the absence of a bound ligand in the apo-form, the water molecules swap places more easily, each of them occupying the sites for a shorter period of time compared to the ligand-bound case simulations.

## Conclusions

We generated and obtained consistent unbinding paths from hM3R for three ligands: tiotropium (**1**) and its analogues **2** and **3**. All three ligands showed similar unbinding paths, including a first rotation of the charged end and a movement of the aromatic rings of the ligand, followed by a dislocation of the tyrosines forming the aromatic cage, finishing with a 90° angle rotation corresponding to the bottleneck while moving toward the receptor tunnel. Therefore, all ligands show a well-defined similar TS position and leave the orthosteric site in a highly homologous mechanism. The main barrier contribution in the unbinding process is known to be related to the ligand leaving the orthosteric site;^18,23,25^ therefore, we did not follow up the subsequent full exit out of the vestibule. Our results support the path described in the study of Kruse et al.^25^ who also found that the charged amine moiety of tiotropium points toward the extracellular space and not toward the orthosteric binding site along its path.^18,25^

We further validated our TS structures by generating unbiased downhill simulations, which allowed us to further analyze the main events driving the unbinding at the TS. Our first Cartesian coordinate-based (XYZ-PCA) data set showed a remarkably good accuracy at predicting the outcome of the simulation at very early times. This first analysis suggested the relevance of the ECL2 loop and the residues at the ends of the transmembrane helices but proved hard to interpret. A more local but high-resolution (3A) data set, which included the relevant protein binding pocket–ligand atomic distances at the TS structure, matched experimentally relevant residues such as D147, Y148, and Y529 and pointed to T231, which is part of the ECL2/TM5 junction. An increased data set (6A) continued to point toward the ECL2/TM5 junction contributions being the most relevant. We further tested the relevance of this region by augmenting our previous 3A data set with these residues (3A + ECL2/TM5). This further justified the key role of the ECL2/TM5 junction. On the other hand, adding, e.g., ECL3 residues to the 3A data set instead did not yield relevant distances from the ECL3 region. This further validated the relevance of the highlighted residues from ECL2/TM5, which also show differences in the protein sequence compared with hMR2 (L225/F181 and E227/N192 substitutions), highlighting potential role in the residence time differences between the two receptors.

Several residues identified by the MLTSA were previously experimentally mutated, further validating their importance in residence time. The available mutations show the largest influence for F224A, Y529A, and N507A in the unbinding kinetics, while D147A, W199A, E227A, K212V, and D517A impact it to a lesser extent. Additional residues we identified here as highly relevant remain yet to be experimentally probed for their role in ligand unbinding kinetics, such as L482, together with the preceding S481, as well as T234 remain to be further studied. Other identified residues that could play a role are C220, I222, L225, S226, and T231.

Our results point to the structural importance of key ligand groups and consistently found specific atoms in the amine end, the carboxyl group, and the thiophene rings to be highly relevant. All three pharmacophore groups match other variants of tiotropium that have a charged end, a middle carboxyl group, and an aromatic ring at the end, either one or two.^18^ Our analysis can therefore provide useful information to propose pharmacophores in future drug design studies for kinetics-based ligand optimization.

To account for all residue interactions with the ligand, a data set with coarser interaction features (allres) was also used. This confirmed the importance of the ECL2/TM5 junction and furthermore pointed to residues at helical ends. Additionally, when the closest ligand–water distances are added to the previous set (allres + wat set), two water molecules also appear at the top. Our results suggest an important role of these molecules, whereby their movement is highly correlated to the ligand entering the orthosteric binding pocket. Additional MD simulations of the apo-form revealed that there are considerably more water molecules inside the orthosteric pocket compared with the ligand-bound states, yet the intracellular and extracellular waters do not connect. In the apo-form, we identified a potassium ion that descended deep into the orthosteric binding pocket similarly to those observed in various experimental structures of A class GPCRs. We hypothesize that such a potassium ion might appear from the intracellular space in the ligand-bound states, yet we did not observe this during our simulations. Both the apo-form and the ligand-bound states showed a very high water occupancy within the orthosteric site, yet the presence of the ligand had a prolongating effect on the residence times of the water molecules.

Importantly, L482 remains to be a top-ranked feature, near a phosphorylation site (S481 for PKG-II)^63,67^ and between two specific binding regions for signaling and activating proteins (SET and PPase 2).^65,66^ We note that more complete initial structures using crystal structures with the kinase domain included and/or with improved resolution might provide further insights into the role of L482 (e.g., in PDB 4U15, the TM6 helix is more ordered). Interestingly, S481 phosphorylation was linked to enhanced dimerization in an allosteric mechanism upon antagonist binding,^68^ proposed to be a general mechanism in the GCPR signal transduction.^69−71^ This suggests that the conformational changes of the ECL2/TM5 junction at the TS crossing transduce a signal across the membrane to the intracellular ICL3 kinase domain of the receptor as the ligand exits or binds the orthosteric site. Our MLTSA analysis appears to capture and identify allosteric effects, opening up potential avenues in various other systems and processes as well,^83,84^ beyond ligand unbinding. Nevertheless, the allosteric signal transduction remains to be studied in more detail, to aid the understanding of the function and mechanism of this biomedically relevant receptor family.

## Supporting Information Available

We provide further supporting information on the MD simulations (analysis of the classical MD, unbinding, and downhill trajectories) and our ML protocols (data set creation, ML models, feature selection) (PDF)

## Abbreviations

MR: muscarinic receptor
GPCR: G-protein-coupled receptor
hMR3: human muscarinic receptor 3
hMR2: human muscarinic receptor 2
COPD: chronic obstructive pulmonary disease
ECL2: extracellular loop 2
TM5: transmembrane region 5
TM6: transmembrane region 6
TM7: transmembrane region 7
ICL3: intracellular loop 3
US: unbound state
BS: bound state
TS: transition state
ML: machine learning
MLTSA: machine learning transition-state analysis
FEP: free-energy profile
MLP: multilayer perceptron
GBDT: gradient boosting decision tree
RAD: relative accuracy drop
RFI: relative feature importance
PCA: principal component analysis
CV: collective variable
MD: molecular dynamics
